# How to Reach the Best Ultrasound Performance in the Delivery Room

**DOI:** 10.1055/s-0042-1759773

**Published:** 2022-12-29

**Authors:** Eduardo Félix Martins Santana, Renata Gomes Castello, Maria Eduarda Tenório Passos, Gabriela Carneiro Freitas Ribeiro, Edward Araujo Júnior

**Affiliations:** 1 Medical Course, Albert Einstein Medical School, São Paulo, SP, Brazil.; 2 Fetal Medicine Unit, Albert Einstein Hospital, São Paulo, SP, Brazil.; 3 Department of Obstetrics, Paulista School of Medicine, Federal University of Sao Paulo, São Paulo, SP, Brazil.; 4 Medical Course, Municipal University of Sao Caetano do Sul, São Paulo, SP, Brazil.

**Keywords:** ultrasound, delivery room, labor, placental disorders, ultrassom, sala de parto, parto, desordens placentárias

## Abstract

Ultrasonography is an instrument that is present in the maternal-fetal assessment throughout pregnancy and with widely documented benefits, but its use in intrapartum is becoming increasingly relevant. From the assessment of labor progression to the assessment of placental disorders, ultrasound can be used to correlate with physiological findings and physical examination, as its benefit in the delivery room cannot yet be proven. There are still few professionals with adequate training for its use in the delivery room and for the correct interpretation of data. Thus, this article aims to present a review of the entire applicability of ultrasound in the delivery room, considering the main stages of labor. There is still limited research in evidence-based medicine of its various possible uses in intrapartum, but it is expected that further studies can bring improvements in the quality of maternal and neonatal health during labor.

## Resumo

A ultrassonografia é um instrumento que está presente na avaliação materno-fetal durante toda a gestação e com benefícios largamente documentados, porém sua utilização no intraparto vem sendo cada vez mais pertinente. Desde a avaliação de progressão de trabalho de parto a avaliação das desordens placentárias, a ultrassonografia pode ser empregada correlacionando com os achados fisiológicos e do exame físico, pois o seu benefício na sala de parto ainda não pode ser comprovado. Há ainda poucos profissionais com treinamento adequado para seu uso na sala de parto e para interpretação correta dos dados. Dessa forma, este artigo tem como finalidade apresentar uma revisão de toda a aplicabilidade do ultrassom na sala de parto, considerando as principais etapas do trabalho de parto. Ainda são limitadas as pesquisas em medicina baseada em evidências sobre os diversos usos possíveis no intraparto, mas espera-se que novos estudos possam trazer melhorias na qualidade da saúde materno-neonatal durante o trabalho de parto.

## Introduction

The use of intrapartum ultrasound has been widely reported as an additional tool for predicting the evolution of successful labor. 1 The sonographic evaluation was not shown to be superior to the vaginal examination (VE), but complementary, as the first is better for the evaluation of head station, position, and caput succedaneum, while cervix dilatation in the active stage of labor (> 4 cm) is better assessed by VE. 2 Sonographic assistance during the first and second stages of labor has the potential to improve labor outcomes, although its real benefits have not yet been proven in large randomized trials. 3 4 5 6 In contrast, the intrapartum Doppler assessment has shown no benefit in perinatal outcomes. 7

Differentiated normal and abnormal sonographic postpartum findings can also be an extra implement for the patients' well-being when the clinical evaluations are doubtful. 8 Despite great acceptability by patients, 9 specially during stressful situations such as prolonged labor (more than 12 hours from the beginning of active phase of the first stage) and unplanned operative delivery, 10 the use of intrapartum ultrasound requires a steep learning curve for good reproducibility; thus, younger obstetricians prefer to rely on clinical and digital examinations, 11 12 even though ultrasound has been proven to be more reliable than VE.

The aim of this article is to present a revision of all ultrasound applicability in the delivery room, considering the main stages of labor.

## Placenta and Cord Anomalies

Placenta and cord anomalies are associated with 30% of intrauterine death risk factors and a high risk of cerebral palsy. 13 Therefore, they are a great cause of concern during prenatal and intrapartum period. The best time to diagnose placental implantations abnormalities is during the second trimester of pregnancy, ideally with a gestational age between 18 and 26 weeks, 14 when is still possible to program the optimum time to perform cesarean section (c-section)—usually around 36 gestational weeks—modifying the neonatal and obstetric outcomes.

The umbilical cord is protected from trauma and compression through the presence of the Warthon jelly and spiraling of blood vessels. 15 Literature has shown that both hypocoiled cords (spiral index below the 10 ^th^ percentile) and hypercoiled cords (spiral index above the 90 ^th^ percentile) are associated with unfavorable neonatal outcomes, 16 17 18 such as higher rates of fetal growth restriction, fetal death, intrapartum fetal heart decelerations, karyotype abnormalities, 19 low birth weight (< 2,500 g), and Appearance, Pulse, Grimace, Activity, and Respiration (APGAR) score < 7 on the 1 ^st^ and 5 ^th^ minutes of life. 15 A prenatal ultrasound assessment of cord coiling is possible; however, no benefit was found in this diagnostic screening since there are no revised means to prevent intrauterine death or a nonreassuring pattern of fetal heart rate in these cases. 20

While 97% of vasa previa cases are diagnosed during prenatal scanning, 12 the benefit of performing the intrapartum diagnosis to foresee possible complications such as maternal bleeding, fetal bleeding, and neonatal death is questioned. Due to the low prevalence of this pathology (0.02–0.27% of all pregnancies), 12 prenatal screening through transvaginal ultrasound becomes unfeasible and is recommended only for women at high risk: *in vitro* fertilization pregnancies, placenta previa, placenta with accessory lobe, velamentous cord insertion, and multiple gestations. 21 22

**Fig. 1:**
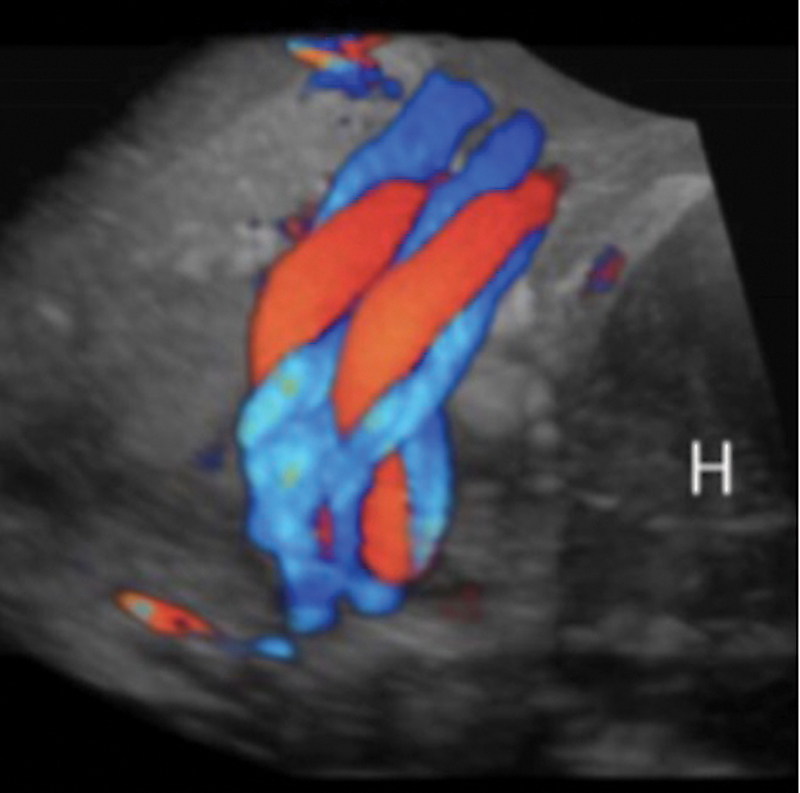
Ultrasound imaging showing multiple nuchal cord loops.

Data in the literature are very vague about intrapartum diagnosis of vasa previa using the Doppler ultrasound, with only two case reports. 23 24 In both cases, the correct diagnostic enables the performance of c-section before the rupture of the vasa previa, with a favorable outcome for the maternal-fetal binominal. Another condition that can lead to risk of maternal and fetal life due to bleeding is placental abruption, present in 0.4 to 1% of all pregnancies. 25 26 The sonographic visualization of retroplacental clots is a finding present in only 15 to 25% of cases and does not interfere with the conduct regarding the interruption of pregnancy, both in term and preterm pregnancies, since maternal and fetal conditions are more important for clinical management. 27 28 The intrapartum ultrasound represents a sensitivity of less than 30% for the diagnosis of placental abruption, and the clinical diagnosis remains the gold standard of this obstetric emergency. 29 On the other hand, the benefit of intrapartum ultrasound use has been proven in relation to the diagnosis of nuchal cord, with a sensibility of 90.2 to 96.8% when using the Doppler mode. 16 30 This finding is present in 22 to 45% of all pregnancies, and it is known that single nuchal cord is not associated with unfavorable perinatal outcomes. 16 31 32 33 However, multiple nuchal cord is associated with worse outcomes, such as perinatal mortality, APGAR score < 7 on the 1 ^st^ and 5 ^th^ minutes of life, fetal distress, and meconium ( Figure 1 ). 16 34 35 36 37

The intrapartum diagnostic of nuchal cord is a good tool in situations of variable deceleration in cardiotocography during labor, as it helps to recognize the cases in which the cardiotocographic pattern is not reassuring due to fetal distress and the cases when the deceleration is due to the presence of nuchal cord. 34 Lastly, the umbilical cord prolapse is a rare situation that affects 0.12 to 0.62% of all pregnancies, with a mortality rate of up to 10% due to compression of the umbilical cord. 20 Some risk factors for this comorbidity are polyhydramnios, prematurity, multiparity, multiple pregnancies, breech presentation, and low birth weight (< 2,500 g). 19 20 38 39 The evident umbilical cord prolapse occurs when the umbilical cord passes between the fetal parts after the premature rupture of membranes and the diagnosis is possible through the VE, while the occult umbilical cord prolapse occurs when the membranes are intact but the cord is ahead of fetal presentation, and the diagnosis is made by ultrasound. 20 The literature has shown low accuracy for the diagnosis of cord prolapse in routine ultrasound, 40 but has shown benefit in the use of transvaginal ultrasound to predict occult umbilical in breech presentation, 41 and the results were better when the occult cord prolapse was previously diagnosed when compared with the evident cord prolapse, suggesting that in high-risk situations, ultrasound evaluation could improve the neonatal outcomes. 42

## Fetal Wellbeing During the Labor

The use of Doppler ultrasound during the labor is still limited for research purposes. However, new studies are emerging, and the application of Doppler is increasingly being studied at this time. Sütterlin et al. 43 evaluated 70 pregnant women in early labor between 38 and 41 weeks of gestation, obtaining Doppler waveforms before and during abnormal fetal heart rate patterns. When an oxygen saturation level of < 30% was maintained for more than 2 minutes, the middle cerebral artery Doppler indices were reversed, indicating morbid fetal hypoxia. These results were considered consistent with the concept that the fetus maintains the oxygen supply to the brain by redistributing blood flow during active labor.

Chainarong and Petpichetchian 44 evaluated the cerebroplacental ratio (CPR) during the labor, and no association was found between CPR and adverse perinatal outcomes with any CPR cut-off values. This study found that fetuses that ended up in a non-reassuring state, necessitating operative delivery, had significantly lower CPR compared with fetuses that did not. Dall'Asta et al. 45 studied the relationship between CPR measured at the beginning of labor and perinatal and delivery outcomes in a cohort of uncomplicated term pregnancies with a single child. The study's conclusion suggests that reduced CPR by itself, although associated with an increased risk of intrapartum distress, represents a poor predictor of adverse perinatal outcomes. Cochrane review assessed the effectiveness of fetal movement monitoring and Doppler ultrasound for the detection and surveillance of high-risk pregnancies and their effect in preventing stillbirths. The combined results of 16 studies showed that the umbilical arterial Doppler assessment in high-risk pregnancies leads to a 29% reduction in perinatal mortality compared with no Doppler assessment. 46 Intrapartum ultrasound (including Doppler) allowed for a greater understanding of the complex physiology of childbirth. Although promising, neither maternal nor fetal intrapartum Doppler has played a role in the true management of intrapartum ultrasound to date. 43

## Labor Progression Through Ultrasound

While digital VE are uncomfortable and subjective exams, 47 with an error rate ranging from 26.6 48 to 33.5% 49 due to interexaminer reproducibility, sonographic measurements are more reliable and could be an additional tool for the evaluation and estimation of a successful labor. 50 Besides, multiple digital VE are associated with ascending infection to the fetus and the uterus 51 52 and are contraindicated in some situations, such as preterm prelabor rupture of membranes and placenta previa. 53

**Fig. 2:**
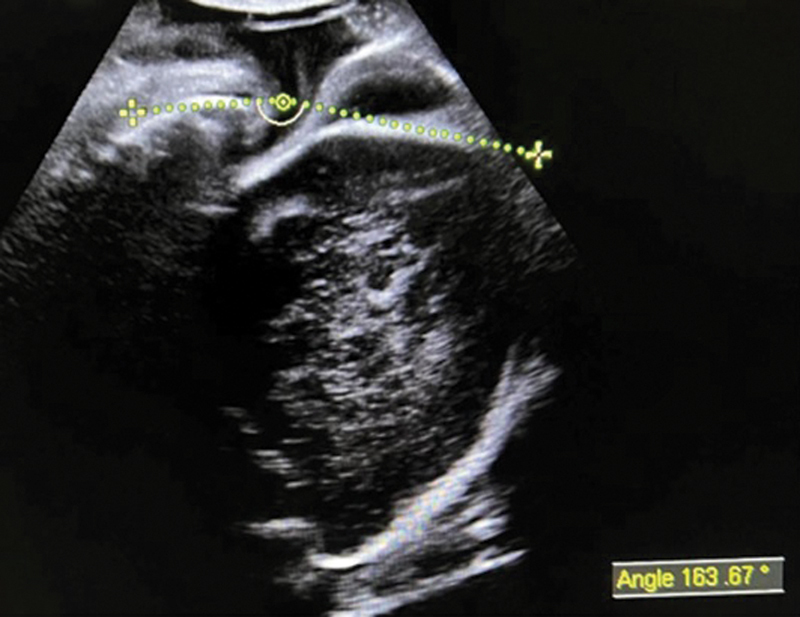
Ultrasound imaging demonstrating the angle of progression (AoP) access.

It is possible to get valuable information that could not be obtained in a VE, such as angle of progression (AoP) which is the angle between a line in the midline of the pubic symphysis and a line running tangentially from the anterior edge of the symphysis to the fetal skull evaluated through transperineal ultrasound ( Figure 2 ); the head progression distance (HPD) which is the shortest distance between the infrapubic line and the leading edge of the fetal skull, also evaluated through transperineal ultrasound; and the head direction (HD) which is the angle between the infrapubic line, perpendicular to the most caudal part of the pubic symphysis, and a line drawn perpendicular to the widest diameter of fetal head, evaluated through abdominal ultrasound. 47 54 The AoP is the most useful measure to predict the success of vaginal delivery, with the manual parasagittal technique being the most reliable, 55 in which the angle is formed between a line drawn along the superior-inferior axis of the pubic bone and a line drawn along the inferior end of the hyperechogenic pelvic bone forming the vertex of the angle with the fetal head.

A systematic review has shown that ultrasound is superior to digital VE for evaluation of fetal head position in the first stage of labor, in addition to the great agreement between the two methods in the assessment of cervical dilatation and a moderate correlation for fetal head station. 48 Although the success rate of digital VE increases with the progression of cervical dilatation, approximately a quarter of digital assessments differ by more than 45° when compared with the sonographic evaluation of fetal head position, 48 49 53 which may lead to unfavorable neonatal outcomes, especially when instrumented deliveries are necessary. 53 Nevertheless, ultrasound evaluation is associated with higher rates of instrumental vaginal delivery, 56 with no difference in maternal and neonatal morbidities when compared with exclusive VE evaluation before operative delivery, 56 57 58 nor in relation to c-section rate, even though ultrasound evaluation corresponds to a greater success in the diagnoses of fetal head position and station. 58

Kameyama et al. 54 described an optimal cut-off from prediction of spontaneous vaginal delivery of 83° for HPD (positive predictive value, PPV = 92.9%), 56 mm for PD (PPV = 94.4%) and AoP of 146° (PPV = 94.3%) right after full cervical dilatation. Ghi et al. 59 have shown that women with spontaneous vaginal delivery had a wider AoP in the begging of second stage of labor (140° ± 20.2°) than the women who had been submitted to operative delivery (122.9° ± 16.7°). Sainz et al. 60 have found that an AoP of 122° ( ± 17.8°) is associated with a complicated operative delivery in nulliparous woman, while an AoP of 149.2° ( ± 15.6°) and a HPD of 50.5 mm are good predictors of uncomplicated deliveries. These facts are consistent with the findings Bultez et al., 61 in which the median of the AoP of 145° is associated with a successful delivery with vacuum extraction, whereas the median of AoP of 136° corresponds to vacuum extraction failure. On the other hand, Kalache et al. 62 described an AoP of 120° as leading to the probability of an easy and successful vacuum or spontaneous vaginal delivery in 90% of the cases.

Chan et al. 63 have shown that parasagittal AoP is an independent predictor for c-section and for non-progression before induction of labor: women with manual parasagittal AoP of 102° (93–111°) and automated parasagittal AoP of 108° (99–115°) were more likely to give birth through vaginal delivery, while women with manual parasagittal AoP of 93° (90–102°) and automated parasagittal AoP of 99° (93–104°) were submitted to c-sections, with no difference between nulliparous and multiparous women. Tse et al. 64 have also shown an additional decrease of 5.28° in the parasagittal AoP and an additional increase of 0.27 cm in HPD for a unit increase in fetal head station and cervical dilation in women requiring c-section, while the additional decrease was 1.35° in the parasagittal AoP and the additional increase was 0.12 cm in HPD in women who achieved vaginal delivery.

Birth weight is an important predictor of neonatal morbidity and mortality and has a strong influence on obstetric and neonatal management. 65 Stubert et al. 66 confirmed that the ultrasound-derived estimated fetal weight during labor at term is an appropriate diagnostic tool, with an average accuracy of 70% within a relative difference of ± 10% to the real birth weight. Furthermore, term-estimated fetal weight has been shown to be unreliable for predicting macrosomia and is therefore not recommended. 66 Considering international guidelines, the cesarean delivery rate should not be higher if fetal weight is estimated immediately before delivery. However, overestimation of fetal weight was associated with an increased risk of c-section. 65 In this study, the increase in the rate of c-section was not accompanied by a decrease in fetal or maternal morbidity. No differences were observed in shoulder dystocia and in third- and fourth-degree perineal lacerations. 66

Yang et al. 67 found that biparietal diameter, abdominal circumference, and estimated fetal weight at 38 weeks of gestation were associated with c-sections for failure to progress in labor after adjusting for confounders. Routine biometry may help identify patients whose intrapartum c-section risk could be reduced by elective induction at 39 weeks. Faschingbauer et al. 65 found that the best results regarding intrapartum estimated fetal weight can be obtained with formulas that use biparietal diameter as the only head measurement. Little et al. 68 suggest that provider knowledge may be associated with a higher rate of c-section; therefore, limiting ultrasound check of fetal weight in the short term may help reduce c-section rate.

**Fig. 3:**
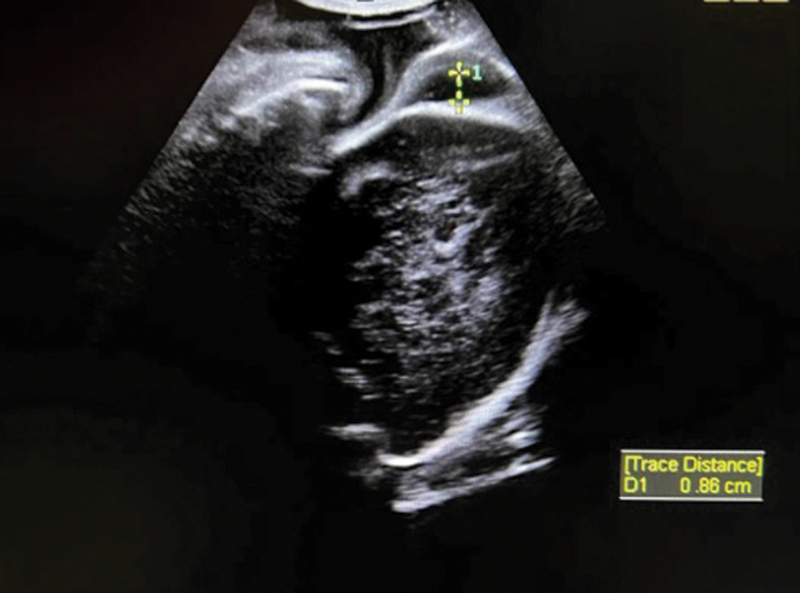
Ultrasound image in the delivery room showing the measurement of caput succedaneum.

**Fig. 4:**
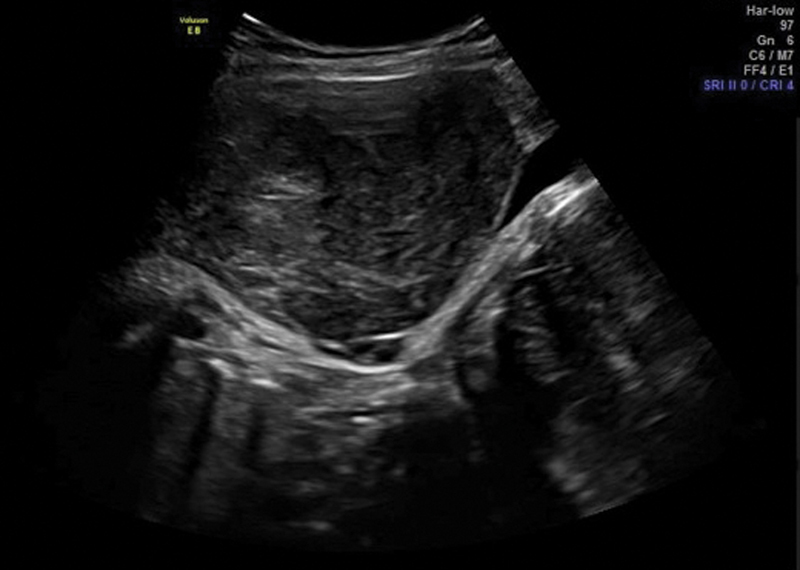
Extensive leiomyoma in the anterior uterine wall.

A different use of ultrasound during the labor is by creating a sonopartogram, which is a conformation of the conventional partogram, with the use of ultrasound parameters of recording assessments during the labor. 69 70 It is possible to evaluate cervical dilatation, fetal head rotation, and fetal head descent, as it is in the conventional partogram, as well as to evaluate caput and molding 69 70 ( Figure 3 ). Although a good agreement was shown between VE and ultrasound evaluation regarding cervical dilatation and head rotation during the first period of labor, the evaluation of head descent was better estimated by VE. 60 Another possibility for the use of ultrasound in the delivery room would be the prediction of success for vaginal delivery on patients with leiomyomas located in regions close to cervix; however, we did not find any data about this topic ( Figure 4 ).

## Ultrasonography at the Immediate Postpartum

Considering postpartum hemorrhage is the leading cause of maternal death worldwide, 71 it is logical to think that ultrasound in the immediate postpartum could be an extra resource to identify possible cases of hemorrhagic complications, including uterine atony, retained products of conception, uterine arteriovenous malformations, and hematomas due birth canal trauma, as well as a good tool for specific treatments, such as curettage, embolization of uterine arteries, and the use of the Bakri balloon. In the immediate postpartum period, the transabdominal approach is preferable for uterus evaluation rather than transvaginal approach. 72

**Fig. 5:**
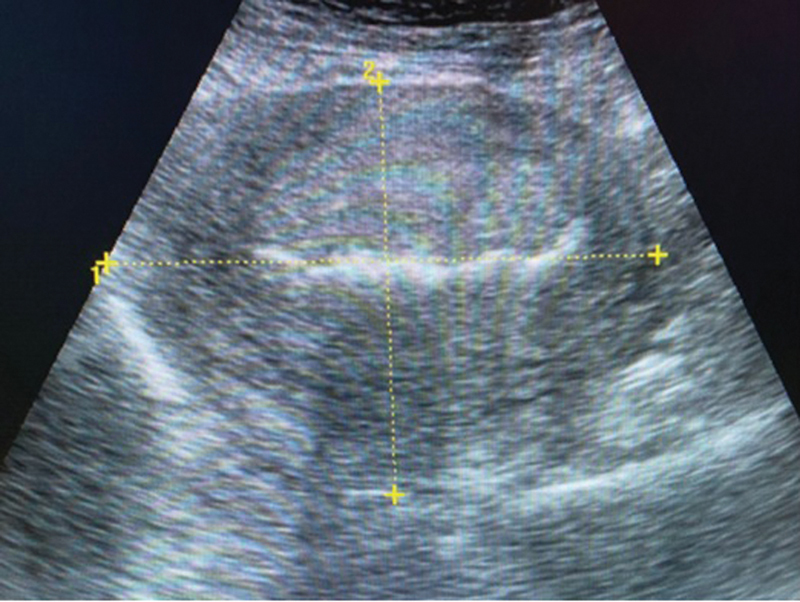
Immediate postpartum ultrasound: endometrial echogenic and uterus size evaluation.

The main cause of postpartum hemorrhage is uterine atony, which can be identified by the loss of at least 500 ml of blood after vaginal delivery or 1000 ml after c-section, associated with the lack of the Pinnard security globe, which represents the uterus involution and can be diagnosed by physical exam, through palpation of the uterine height. 73 74 Through sonographic exam, the mean uterine length is 16.1 ± 1.7 cm, 75 while the maximum anterior-posterior uterine dimeter reported was 9.2 cm 76 ( Figure 5 ). Concerning endometrial evaluation, some studies have shown no correlation between the duration or amount of bleeding and the presence of echogenic material diagnosed by postpartum ultrasound. 75 77 78 79 A recent systematic review has found that the upper limit for endometrial thickness (95 ^th^ centile) measured by abdominal ultrasound within 24 hours postpartum is 22 mm, 80 with no statistically significant difference between vaginal delivery or c-section, or between nulliparous and multiparous women. 77 80

Another possible cause of postpartum bleeding is the presence of retained placental tissue, which can happen in approximately 1% of term deliveries. 81 The literature has shown a variable sensitivity (42–94%) and specificity (62–92%) for the use of ultrasound in the uterus evaluation after placental removal. 82 The gray scale itself is not the best option as a diagnostic method, as the appearances of retained placental tissue in the immediate postpartum are highly variable and can be represented as echogenic mass, heterogeneous mixed density mass, and normal endometrial cavity. Therefore, it could not be correlated with a need for intervention and might not change patient outcomes. 81 82 The identification of thickened endometrial echo complex > 10 mm, associated with vascular flow detection on color Doppler, is highly suggestive for retained placental tissue. 83 However, a hypervascular area can be physiologic in the postpartum period and disappear spontaneously or after removal of placental remnants 84 85 and, therefore, there is no gold standard protocol for diagnosing retained placental tissue through imaging exams. 86

As for the placenta accreta spectrum, the diagnostic should preferably happen prenatally, so the best time and place for delivery can be arranged, as an intraoperative hysterectomy might be necessary. 87 However, when there is no prenatal diagnosis and the patient goes through labor, the diagnostic is made during the third stage of labor, which may lead to major bleeding. 87 88 The normal placental separation can be sonographically characterized by decreased blood flow while the placenta is detaching from the myometrium, whereas the presence of placenta accreta spectrum can be sonographically characterized by the persistent blood flow between the placenta and the myometrium. 88

A rare, but serious situation of postpartum hemorrhage, is the uterine rupture. Its prevalence is less than 1% after a vaginal delivery after one c-section, increasing to up to 2% when the vaginal delivery occurs after more than two previous c-sections. 89 It should be suspected in patients with vaginal delivery after c-section or any uterine surgery, presenting postpartum hemorrhage and hypovolemic shock. 90 The diagnostic must be done as soon as possible, and a transabdominal ultrasound would show an echo-free space or mass lesions, possibly corresponding to intraperitoneal bleeding or retroperitoneal hematoma. 90

Lastly, an unusual but possible cause of hemodynamic instability in the delivery room is the presence of a vulvar or paravaginal hematoma. 91 92 This complication might happen specially after direct injury of the perineum, from instrumental deliveries, vaginal laceration, or episiotomy. 92 The main symptoms are pelvic and perianal pain, swelling of the vulva, paravaginal mass, and urinary retention due mechanical urethral obstruction. 91 92 The use of transperineal or transabdominal ultrasound can provide precise information about the presence, location, and size of the vaginal hematoma, with similar results findings with computed tomography, but with the advantage that it can be performed in the labor ward, immediately after delivery. 91

## Conclusion

The performance of ultrasound in the delivery room is still a poorly explored resource in maternity hospitals. However, with the potential to improve the diagnosis and interpretations of situations and allow for more timely interventions, since it is a tool with the potential to complement (and not replace) clinical practice. There is still little evidence-based medical research on the several possibilities of its intrapartum use, but we expect that further studies could provide improvements in the quality of maternal-neonatal health during the labor.
